# Supplementary material for: Pathological Role of Pin1 in the Development of DSS-Induced Colitis
Source: Cells. 2021 May 17;10(5):1230. doi: 10.3390/cells10051230 (PMC8155908; doi:10.3390/cells10051230)
Supplement: Supplementary file 1 [file cells-10-01230-s001.zip › cells-1196910-supplementary.pdf]

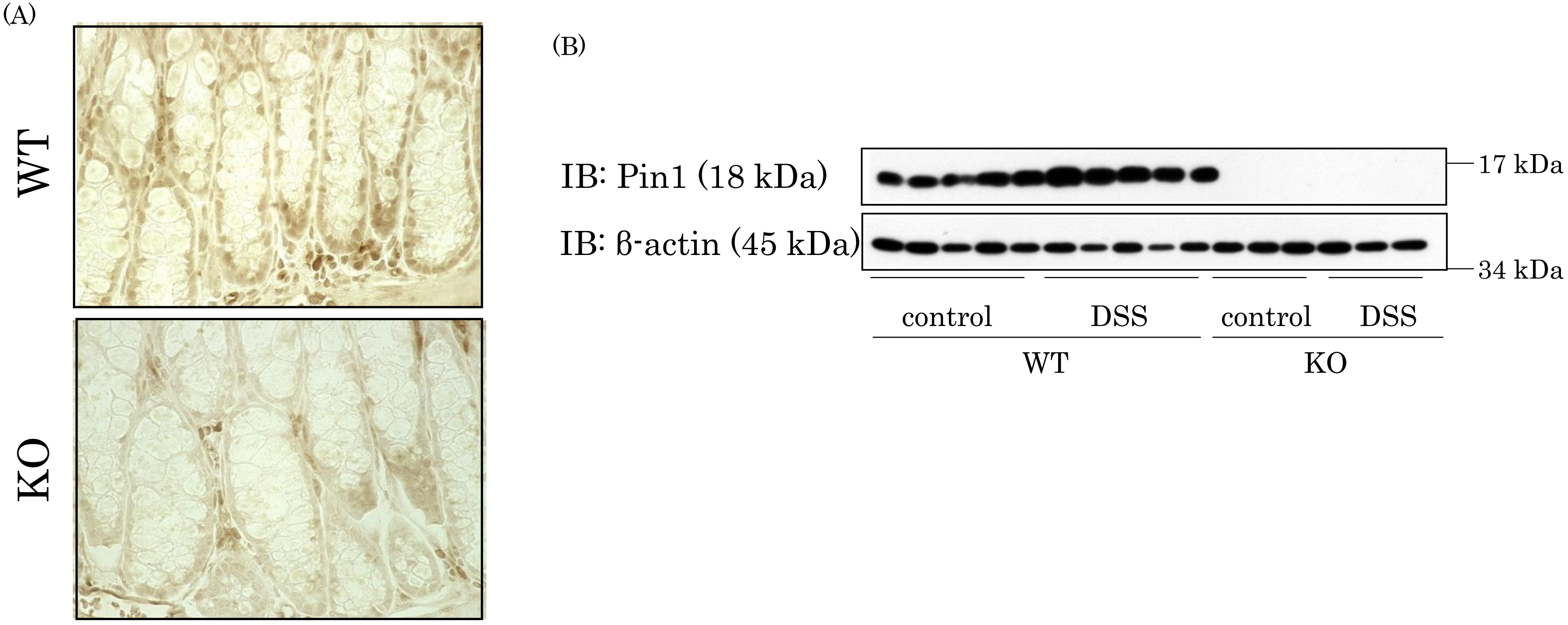

Supplementary Fig.1 Pin1 proteins in the colon of KO mice were deleted
(A) Colon sections were stained with Pin1 antibody
(B) Pin1 proteins in the colon were detected by western blotting
